# Effect of β-Mannanase Supplementation on Growth Performance, Ileal Digestibility, Carcass Traits, Intestinal Morphology, and Meat Quality in Broilers Fed Low-ME Diets

**DOI:** 10.3390/ani12091126

**Published:** 2022-04-27

**Authors:** Muhammad Umar Yaqoob, Muhammad Yousaf, Muhammad Issa Khan, Minqi Wang

**Affiliations:** 1 Key Laboratory of Animal Nutrition and Feed Science (Eastern China), Ministry of Agriculture, College of Animal Science, Zhejiang University, Hangzhou 310058, China; 11817048@zju.edu.cn; 2 Institute of Animal and Dairy Sciences, University of Agriculture, Faisalabad 38000, Pakistan; yousaf@uaf.edu.pk; 3 National Institute of Food Science and Technology, University of Agriculture, Faisalabad 38000, Pakistan; drkhan@uaf.edu.pk

**Keywords:** β-mannanase, broilers, carcass traits, digestibility, growth performance, intestinal morphology

## Abstract

**Simple Summary:**

β-mannans, an example of non-starch polysaccharides, is one of the important anti-nutritional factors found in many leguminous feedstuffs used as raw material in poultry feed formulation. β-mannans bind to nutrients, reduce their bioavailability, and also increase the viscosity of intestinal digesta, which prevents the proper mixing of enzymes with digesta and affects the digestibility of nutrients. All these conditions ultimately reduce the growth performance of poultry birds. So, the objective of present study was to lower the effect of β-mannans by using β-mannanase in broiler diets. Results suggested that β-mannanase supplementation improved the intestinal morphology, which helped to increase the dry matter and crude fiber digestibility in broilers fed low energy diets. In addition, growth performance of broilers was not affected by reducing dietary energy levels with added β-mannanase than the Control group. Similarly, carcass traits were not affected by decreasing dietary energy levels with supplementation of β-mannanase. In conclusion, it could be stated that a low caloric diet with added β-mannanase had no negative effect on growth performance and carcass characteristics; however, it improved the dry matter and crude fiber digestibility.

**Abstract:**

Experiment was designed to analyze the effect of low caloric diets, supplemented with β-mannanase on growth performance, carcass characteristics, nutrient digestibility, and other parameters in broilers. In this study, 400 broiler chicks were randomly divided into four treatments (Cont: without β-mannanase; LM-30, MM-60, and HM-90: supplemented with 200, 400, and 600 mg/kg β-mannanase, respectively). Dietary metabolizable energy (ME) in Cont was standard (starter diet 3100 kcal/kg; finisher diet 3200 kcal/kg) and reduced by 30, 60, and 90 kcal/kg, correspondingly in β-mannanase-supplemented treatments. The results exhibited that growth performance was not affected by reducing dietary energy levels with supplementation of β-mannanase. Ileal digestibility of DM and CF was improved (*p* < 0.01) by supplementation of β-mannanase at low dietary ME levels. Concerning carcass traits, the relative weight of breast meat, drumstick, and abdominal fat increased (*p* < 0.05) with β-mannanase supplementation in low-ME diets. Treatment HM-90 showed significantly (*p* < 0.05) better results regarding villus height and VH:CD. It could be concluded from the present results that supplementation of β-mannanase could improve the nutrient digestibility so that it is possible to reduce the dietary energy level without compromising production performance, carcass trails, and meat quality in broilers.

## 1. Introduction

Despite the great advances in poultry nutrition, there is a need to identify factors that reduce nutrient bioavailability and negatively affect the poultry production. Among the many anti-nutritional factors, non-starch polysaccharides (NSP) are of great interest because they are present in many poultry feed ingredients and reduce the digestibility and bioavailability of nutrients. Cellulose, hemicellulose, arabinan, mannan, and xylan are NSP that reduce or prevent nutrient utilization in poultry [1]. β-mannans are a group of specific types of hemicelluloses that occur in two forms, glucomannans, and galactomannans, and are the most abundant polysaccharide in nature after xylan. The most important enzyme after xylanases are mannanases [2], which are used for the hydrolysis of hemicellulose. Many leguminous feedstuffs, including soybeans, contain significant amounts of β-mannans [1], which are considered harmful anti-nutritional factors in poultry diets [3]. β-mannans have been shown to negatively affect animal health and growth by reducing nutrient digestibility, resulting in lower feed efficiency [4], and also reducing water and glucose absorption. They are also responsible for increasing the viscosity of intestinal digesta and disrupting the gut micro-ecology. These problems could be solved by using exogenous fiber-degrading enzymes, such as β-mannanase, β-glucanase, and protease, in poultry diets. β-mannanase decreases the viscosity of intestinal digesta and helps in the release of bound nutrients, such as D-mannose, which serves as an energy source [5]. They also reduce the population of pathogenic bacteria in the intestine. It has been reported that β-mannanase improves immunity and reduces energy depletion in an unproductive manner [6] and increases the availability of energy to animals. Similarly, the beneficial effect of supplementation with β-mannanase in broilers [5,7], turkey [3], and laying hens [8] has been reported by many researchers. β-mannanase significantly improved feed conversion efficiency by degrading carbohydrates resistant to endogenous enzymes [5,8]. Considering the importance of β-mannanase supplementation and the presence of β-mannan in the corn–soybean diet, the present study was designed to investigate the effects of varying levels of β-mannanase supplementation in energy gradient-reduced diets on the growth performance, nutrient digestibility, carcass characteristics, and meat quality in broilers.

## 2. Materials and Methods

### 2.1. Animals and Experimental Design

Feeding trial was conducted on 400 broiler chicks (Arbor Acres) at the R&D farm, Five Star Feeds Pvt. Ltd. in Gujranwala, Pakistan. The temperature in the house was maintained at 35 °C during the brooding period of the first week, and reduced by 5 °C per week and maintained at 21 °C. The relative humidity was maintained at 65% and stocking density of 0.65 ft^2^ per bird was practiced. During the first week, 23 h of light and a 1 h dark period was practiced, followed by 19 h of light and a 5 h dark period.

During the first week, chicks were fed on commercial starter diet with crude protein level of 23% and ME 3000 kcal/kg. At the start of the second week, chicks were randomly divided into twenty replicates, and fiver replicates were designated to one treatment, while the number of chicks per treatment was 20. The experimental design is shown in Table 1.

The formal feeding trial extended over 4 weeks. During the first two weeks, chicks were fed on grower diets (grower phase 8–21 days) and during the subsequent two weeks they were fed on finisher diets (finisher phase 22–35 days). All diets were formulated according to feeding standard of Arbor Acres Broiler Nutrition Specification 2019, as showed in Table 2 and Table 3. Chicks in the different groups were fed iso-nitrogen and gradient-reduced (30 kcal/kg) metabolizable energy (ME), without (Cont), or with various amounts of supplemental β-mannanase (low β-mannanase: LM, 200 mg/kg; medium β-mannanase: MM, 400 mg/kg; high β-mannanase: HM, 600 mg/kg), respectively. ME of diets supplemented with LM, MM, or HM were reduced by 30, 60, and 90 kcal/kg, respectively, and the corresponding treatments were designated LM-30, MM-60, and HM-90, respectively.

Hemicell^®^ (Elanco, Greenfield, IN, USA) was used as a source of β-mannanase with an activity of approximately 160 × 10^6^ U/kg. Animals had free access to feed and water throughout the experiment. All management and biosecurity conditions were strictly practiced throughout the experiment.

### 2.2. Samples Collection and Measurements

#### 2.2.1. Growth Performance

To determine the effect of dietary treatments on growth performance, feed intake and weight gain were recorded on weekly bases. From the data of feed intake and weight gain, the feed conversion ratio (FCR) was calculated by using the following relationship:

$$\mathrm{FCR}=\frac{\mathrm{Feed}\;\mathrm{inatke}\;\mathrm{in}\;\left(g\right)}{\mathrm{Weight}\;\mathrm{gain}\;\left(g\right)}$$

Data of feed intake, weight, and FCR were compiled and presented as the starter phase, finisher phase, and overall data.

#### 2.2.2. Digestibility Trial

The Celite^®^ was supplemented in the last five days of feed at the level of 1%, to determine nutrient digestibility. Twenty animals (one animal per replicate) were selected randomly at the end of feeding trial (35 day-old boilers) and slaughtered to obtain ileal contents for determination of nutrient digestibility. After collection, digesta was immediately placed in plastic bags and preserved at −10 °C for further analysis. To determine the dry matter (DM) content, crude protein (CP), crude fiber (CF), and ether extract (EE) in ileal contents and feed samples, proximate analysis was performed (AOAC, 2000). The digestibility coefficient was calculated using the following Equation:

$$\mathrm{Digestibility}\;\mathrm{coefficient}\;\left(\%\right)=100-(100\times \frac{\mathrm{Marker}\;\mathrm{in}\;\mathrm{feed}\;\left(\%\right)}{\mathrm{Marker}\;\mathrm{in}\;\mathrm{ileal}\;\mathrm{contents}\;\left(\%\right)}\times \frac{\mathrm{Nutrient}\;\mathrm{in}\;\mathrm{ileal}\;\mathrm{contents}\;\left(\%\right)}{\mathrm{Nutrient}\;\mathrm{in}\;\mathrm{feed}\;\left(\%\right)})$$

#### 2.2.3. Carcass Characteristics

After the feeding experiment, twenty animals (one animal per replicate) were selected randomly and slaughtered to access the effect of treatments on carcass traits. To calculate the dressing percentage, live body weight and carcass weight were recorded. The weight of all carcass parts (thigh, breast, drumstick, and wing; weight of only one side), abdominal fat, and internal organs (liver, heart, and gizzard) was recorded and expressed as a percentage of carcass weight.

#### 2.2.4. Intestinal Segments and Morphology

Intestinal segments were separated to measure the length and weight of each part. Jejunum was cut from midsection and about a 2 cm piece was fixed in neutral buffered formalin (10%) for three weeks to prevent any postmortem changes. The composition of neutral buffered formalin was as follows: 50 mL formalin (73%, *w*/*v*); 450 mL distilled water; 2 g/L sodium dihydrogen phosphate; 3.3 g/L disodium hydrogen phosphate. Slides of jejunal segments were prepared by dehydrating and then embedding the specimens in paraffin. Specimen were then sectioned (5 μm) with a microtome and stained with hematoxylin and eosin. Morphometry was performed according to the previous method [9] using a light microscope with a computerized morphometric system (Nikon Corporation, Tokyo, Japan).

#### 2.2.5. Meat Quality Analysis

Whole breast and thigh parts were collected from each slaughtered animal for meat quality analysis. The method of Jang et al. [10] was used to determine the water holding capacity (WHC) of breast and thigh meat samples. Samples were centrifuged at 5000 rpm (Eppendorf Centrifuge 5804R, Taufkirchen, Germany) for 10 min at 4 °C to determine WHC.

The cook loss of meat samples (both breast and thigh) was determined by cooking the samples in a hot water bath (90 °C) for half an hour followed by cooling the samples to room temperature. The initial weight (W1) was recorded and the weight loss after cooking and removal of water was also recorded (W2). Cook loss was calculated using the following combination:

$$\mathrm{Cook}\;\mathrm{loss}\;\left(\%\right)=\frac{W1-W2}{W1}\;\times 100$$

Shear force of breast meat fibers was determined using a texture analyzer, through Penetrometry (LAMY Rheology, Texture Analyzer TX-700, Champagne au Mont d’Or, France). During this analysis, the blade of the texture analyzer was set perpendicular to cooked breast muscle fibers. The following parameters were set on the apparatus: measurement time: 10 s; maximum speed: 1 mm/s; starting force: 2 g; waiting position: 10 mm; maximum distance 50 mm and set force: 1000 g.

A slurry was prepared using 10 g of a meat sample in 90 mL of distilled water to determine the pH of meat samples. Samples were homogenized using VELP Scientifica OV5, Usmate Velate, Italy. A portable pH meter (HI 99163) from Hanna Instruments, Inc., Woonsocket, RI, USA, was used to measure the pH of slurry in duplicate.

Meat samples (both breast and thigh) were processed through a meat colorimeter (Precision colorimeter, STPR45, Shenzhen, China) to analyze the color of samples and each sample was analyzed form three different positions. Before using the instrument, it was calibrated using black and white calibration tiles following the manufacturer’s instructions. From the colorimetric analysis of the meat samples, the following values were recorded CIE L, a and b.

### 2.3. Statistical Analysis

Data collected for all parameters were subjected to one-way analysis of variance using a general linear model in SPSS 26. To determine the statistical difference between the groups, Tukey’s test was performed and the level of significance was set at *p* < 0.05. Data for all parameters were presented as mean and standard error of the mean (SEM).

## 3. Results

### 3.1. Growth Performance

The results showed that growth parameters were not affected (*p* > 0.05) by dietary treatments (Table 4). During the starter phase, numerically highest feed intake (1012.42 g) and weight gain (740 g) were observed in MM-60, with 1.37 FCR, while lowest FCR was observed in LM-30 (1.34). During the finisher phase, the numerically highest weight gain (1272.41 g) and feed intake (2178.96 g) were observed in Cont; however, better FCR was observed in MM-60 (1.67). Overall data showed that all parameters of growth performance remained unaffected by reducing dietary energy levels with β-mannanase supplementation in broilers.

### 3.2. Ileal Digestibility

**Figure 1:**
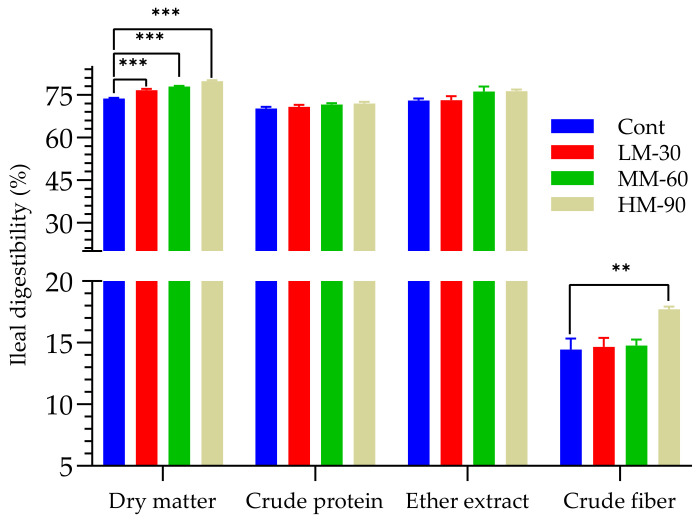
Effect of β-mannanase supplementation on ileal digestibility of nutrients in broilers fed low energy diets. Cont (ME 3100 kcal/kg): without β-mannanase; LM-30 (ME −30 kcal/kg), MM-60 (ME −60 kcal/kg) and HM-90 (ME −90 kcal/kg) were supplemented with 200, 400, and 600 mg/kg β-mannanase, respectively. Statistical difference from corresponding control value for a given parameter is annotated by: ** *p* < 0.01, *** *p* < 0.001.

A significant (*p* < 0.01) effect of dietary treatments was observed on ileal digestibility of DM and CF (Figure 1). Lowest ileal digestibility of DM was observed in the Cont (73.69%), which was significantly (*p* < 0.001) increased up to 79.82% in HM-90 treatment (ME −90 kcal/kg; 600 mg/kg β-mannanase). Similarly, the highest ileal digestibility of CF (17.72%) was observed in the HM-90 treatment, which was about 3% higher than that of the Cont. In addition, the highest (*p* > 0.05) ileal digestibility of other nutrients (CP 71.93%; EE 76.30%) was also observed in the HM-90 treatment.

### 3.3. Carcass Characteristics

**Figure 2:**
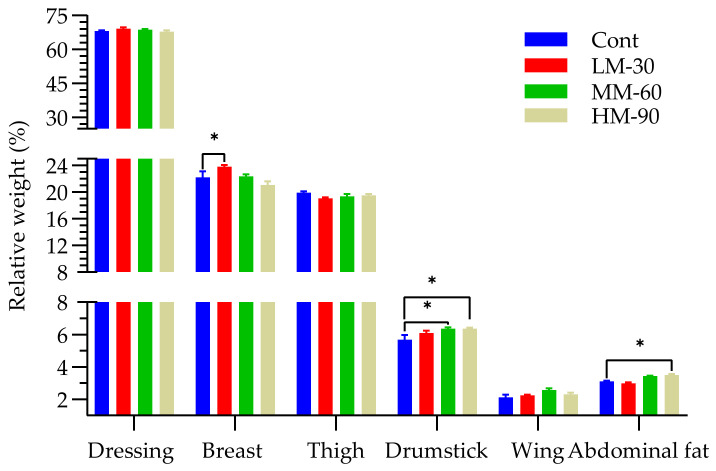
Effect of β-mannanase supplementation on dressing percentage and carcass parts in broilers fed low energy diets. Dressing is dressing percentage, while parts are expressed as a percentage of carcass weight. Cont (ME 3100 kcal/kg): without β-mannanase; LM-30 (ME −30 kcal/kg), MM-60 (ME −60 kcal/kg), and HM-90 (ME −90 kcal/kg) were supplemented with 200, 400, and 600 mg/kg β-mannanase, respectively. Statistical difference from corresponding control value for a given parameter is marked with *: *p* < 0.05.

The results in Figure 2 showed that dietary treatments had a positive effect (*p* < 0.05) on relative weight of the breast and drumstick, whereas the relative wing weight was only slightly affected (*p* = 0.067). The highest carcass weight was observed in Cont (1436 g) and the lowest in HM-90 (1368 g). Carcass weight was decreased by reducing the dietary energy levels, it was 1386 g in LM-30 and it decreased to 1372 g in the MM-60 treatment. The highest values of dressing percentage (69.14%) and breast meat yield (23.84%) were found in LM-30. Relative weight of the drumstick (%) of MM-60 and HM-90 was similar and higher than that of Cont. Relative abdominal fat percentage was increased (*p* < 0.05) by β-mannanase supplementation in broilers and the highest abdominal fat percentage (3.5%) was found in HM-90.

### 3.4. Internal Organ Indexes

**Figure 3:**
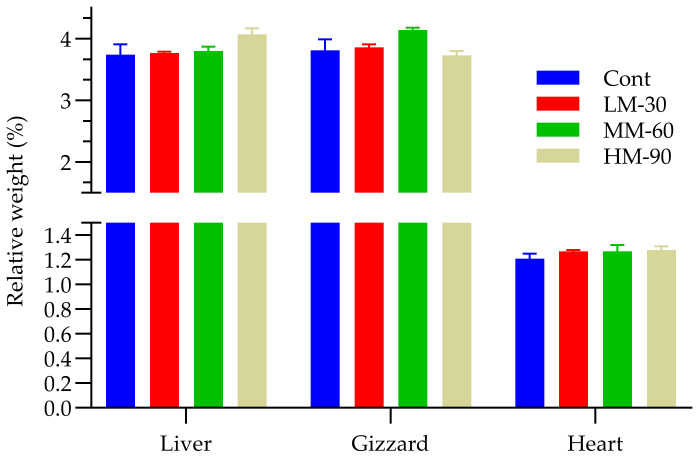
Effect of β-mannanase supplementation on organ indexes in broilers fed low energy diets. Cont (ME 3100 kcal/kg): without β-mannanase; LM-30 (ME −30 kcal/kg), MM-60 (ME −60 kcal/kg) and HM-90 (ME −90 kcal/kg) were supplemented with 200, 400, and 600 mg/kg β-mannanase, respectively.

The relative weight of internal organs (heart, liver, and gizzard) was not affected by lowering the energy contents of the diet with the addition of β-mannanase (Figure 3). The highest relative weight of the heart (1.28%) and liver (4.07%) was found in HM-90 and lowest in Cont (heart 1.21%; liver 3.74%).

### 3.5. Intestinal Segments and Morphology

**Figure 4:**
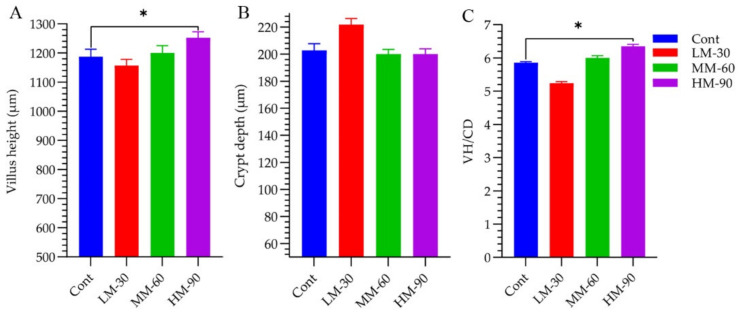
Effect of β-mannanase supplementation on intestinal morphology in broilers fed low energy diets. (**A**): villus height; (**B**): crypt depth; (**C**): villus height to crypt depth ratio. Cont (ME 3100 kcal/kg): without β-mannanase; LM-30 (ME −30 kcal/kg), MM-60 (ME −60 kcal/kg) and HM-90 (ME −90 kcal/kg) were supplemented with 200, 400, and 600 mg/kg β-mannanase, respectively. The statistical difference from corresponding control value for a given parameter is indicated by: * *p* < 0.05.

**Figure 5:**
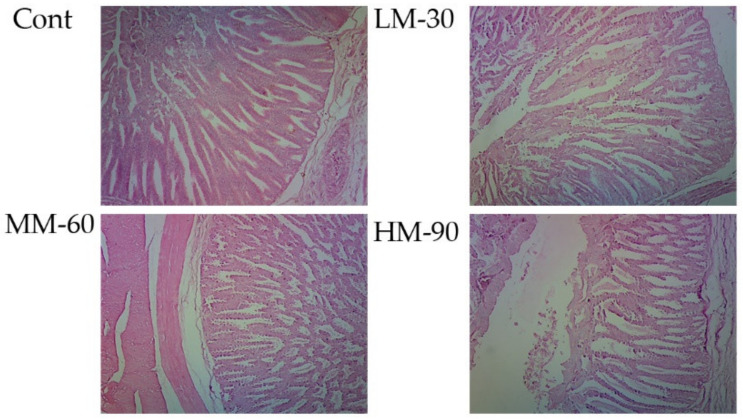
Photomicrographs showing the effect of β-mannanase supplementation on jejunum morphology in broilers fed low energy diets. Cont (ME 3100 kcal/kg): without β-mannanase; LM-30 (ME −30 kcal/kg), MM-60 (ME −60 kcal/kg), and HM-90 (ME −90 kcal/kg) were supplemented with 200, 400, and 600 mg/kg β-mannanase, respectively. H&E staining, the scale is 10×.

Significant effects (*p* < 0.05) of dietary treatments were found on the weight and length of some intestinal segments (Table 5). The weight of the duodenum was lower in the LM-30 treatment than in the Cont treatment (*p* < 0.01). Similarly, the weight of the ileum was lower in treatment LM-30 (*p* < 0.01) and MM-60 (*p* < 0.001), whereas it remained unaffected in treatment HM-90. Regarding the length of intestinal segments, the length of jejunum was longer in all treatments than that in Cont treatment (*p* < 0.001). However, the length of ileum was decreased in both MM-60 (*p* < 0.01) and HM-90 (*p* < 0.001) compared to the Cont treatment. The weight and length of cecum were unaffected by dietary treatments. The results showed that the morphology of the jejunum was significantly improved by using the highest supplemental level of β-mannanase (HM-90) (Figure 4). Villus height and villus height to crypt depth ratio in HM-90 were significantly higher than that of the Cont group. Microscopy analysis revealed normal long intestinal villus and crypt depth in Cont. Increased villus width and crypt depth with mild to moderate cellular infiltration was observed in LM-30. MM-60 showed normal intestinal villus length and crypt depth with mild cellular infiltration. HM90 showed increased villus length and normal crypt depth with mild necrosis (Figure 5).

### 3.6. Meat Quality

The results of meat quality-related parameters showed that they were not affected by the different treatments, except pH after 24 h, which was increased in both breast (*p* < 0.05) and thigh meat (*p* < 0.01) (Table 6), with the lowest pH of thigh (6.07) and breast meat (5.89) in Cont. The highest value of shear force (1025.78 g) for breast meat was observed in Cont, followed by HM-90 (1012.13 g), and the lowest was observed in MM-60 (874.88 g). The lowest cook loss for breast (25.56%) and thigh meat (31.91%) was observed in MM-60 and Cont, respectively. The WHC value was also not affected by dietary treatments (*p* > 0.05). WHC of breast meat varied from 44.75 to 46.27% (Cont vs. LM-30) and that of thigh meat from 41.82 to 44.15% (Cont vs. MM-60). The light color (CIE L) of breast meat was affected by dietary treatments (*p* = 0.022), and its value was lower in HM-90 than in Cont (*p* < 0.05). The yellow and red color (CIE a, b) of the breast and thigh meat samples were not affected by the dietary treatments.

## 4. Discussion

Several feed ingredients, especially soybean, have some contents of β-mannans, which affect the availability and utilization of nutrients. Therefore, the addition of β-mannanase could improve the utilization of nutrients form soybeans. The results showed that low ME diets enriched with β-mannanase had no effects on the growth performance of broilers during the study period of this experiment. These findings are supported by Hussein et al. [11], stating that growth performance of the broilers are not compromised by reducing the ME levels in the case of enzyme supplementation. They further stated that enzyme supplementation has a critical role in partially replacing the energy from poultry diets. Naqvi and Nadeem [12] also found similar results by using low-ME diets with a blend of enzymes (blend of xylanase, beta glucanase, and cellulase). In addition, no effect of dietary supplementation of xylanase or amylase was found on ADG, ADFI, and FCR of broilers [13]. Comparable growth performance of broilers fed low-ME, enzyme supplementation diets with the Control might be due to the availability of more energy from the nutrients and reduced viscosity of intestinal contents, which improve the absorption of nutrients [14]. In addition, β-mannanases might have a role in reducing the negative effect of NSP on the corn–soybean diet [15].

Nutrient digestibility significantly improved by β-mannanase supplementation in a low calorie diet and the results are consistent with previous literature that DM digestibility [16,17] was significantly improved with enzyme supplementation. As with previous studies, positive effects of dietary treatments were also found on CP digestibility [18]. Exogenous enzyme supplementation decrease digesta viscosity and increase the digestibility of DM and N in broilers [19]. β-mannanase might increase the activity of digestive enzymes, and improvement in nutrient digestibility might be due to the fact that enzymes break down the cell wall and help to release the nutrient contents by reducing its integrity [20].

In general, dietary treatments also affected the morphology of different segments of the gastrointestinal tract compared to the Control. Similar results were found by Wang et al. [21] stated that enzyme supplementation decreases the weight and length of ileum. Further, Brenes et al. [22] supported these results that enzyme supplementation reduces the length of the duodenum and ileum. The difference in the digestive tract structure and functions might be due to difference in the viscosity of intestinal digesta due to NSP contents in enzyme supplemented and non-supplemented groups. In view of the above-mentioned findings, it could be concluded that supplementation of enzymes altered the morphology of different parts of the digestive tract. In the present study, jejunal morphology improved by increasing the dietary β-mannanase levels in low energy diets. Treatment HM-90, showed significantly (*p* < 0.05) better villus height and VH:CD. For nutrient absorption, the small intestine is a main part of the digestive tract. Intestinal morphological parameters, such as crypt depth, villus height, and goblet cell are affected by the dietary manipulation especially from the enzyme supplementation [23]. Poor nutrient absorption could be caused due to shorter villus and deeper crypts resulting in increased secretion of water and electrolytes in the GI tract and, thus, compromised performance [9]. Yet, despite lower calorie content, enzyme supplementation seems to improve nutrient bioavailability by enhancing digestion and improving small tract morphology.

Mohammadigheisar et al. [24] reported that most of the meat quality traits were not affected by enzyme supplementation, which is consistent with the present study. Bin [25] also suggested that there is no connection between commercial enzyme supplementation and meat quality. In the present study, only a lower CIE L* value of breast meat was found in HM-90, CIE a, b of breast meat and the thigh meat color were not affected by dietary treatments, which is consistent with Smith et al. [26], who reported that supplemental enzymes resulted in lighter breast meat color but leg meat color was not affected. However, Hussein et al. [11] reported contrary results regarding enzyme supplementation on meat color. Actually, meat color is not only affected by diet but also influenced by many other factors, including pH value, myoglobin content, total haem content, age, sex, and breed of the bird.

## 5. Conclusions

The results obtained from the present study indicated that reducing dietary energy combined with β-mannanase supplementation exhibited no negative effect on growth performance of broilers during grower and finisher phases. β-mannanase improved the nutrient digestibility so that it was possible to reduce the dietary energy level without compromising production performance, carcass trails, and meat quality in broilers.

## Figures and Tables

**Table 1:** Details of experimental groups.

Treatments	Chicks: Replicates	ME kcal/kg		β-Mannanase
		Starter Phase	Finisher Phase	
T1: Cont	n = 100; R = 5	3100	3200	-
T2: LM-30	n = 100; R = 5	3070	3170	200 mg/kg
T3: MM-60	n = 100; R = 5	3040	3140	400 mg/kg
T4: HM-90	n = 100; R = 5	3010	3110	600 mg/kg

**Table 2:** Ingredient composition and nutrient profile of grower feeds (8–21 days).

Ingredients (%)	Treatments			
	Cont	LM-30	MM-60	HM-90
Corn	55.67	56.32	56.98	57.59
Soya bean meal	27.79	27.67	27.56	27.44
Canola meal	8.00	8.01	7.99	8.00
Soybean oil	5.04	4.48	3.92	3.36
Monocalcium phosphate	0.74	0.74	0.74	0.74
Limestone	1.24	1.24	1.24	1.24
L-Lysine HCl	0.38	0.38	0.39	0.39
DL-Methionine	0.27	0.27	0.27	0.26
Threonine	0.08	0.08	0.08	0.08
β-mannanase	-	0.02	0.04	0.06
Sodium chloride	0.25	0.25	0.25	0.25
NaHCO_3_	0.23	0.23	0.23	0.28
Phytase	0.01	0.01	0.01	0.01
VitaMin Premix *	0.30	0.30	0.30	0.30
Total	100	100	100	100
ME (Kcal/kg)	3100	3070	3040	3010
Crude protein	21.50	21.50	21.50	21.50
Lysine	1.29	1.29	1.29	1.29
Methionine	0.51	0.51	0.51	0.51
Calcium	0.87	0.87	0.87	0.87
Available P	0.44	0.44	0.44	0.44

Cont (ME 3100 kcal/kg): without supplemental β-mannanase; LM-30 (ME −30 kcal/kg), MM-60 (ME −60 kcal/kg) and HM-90 (ME −90 kcal/kg) were supplemented with 200, 400, and 600 mg/kg β-mannanase, respectively. * Vit. A 15,000 IU/kg, D3 3000 IU/kg, E 60 IU/kg, K3 3 mg/kg, B1 2 mg/kg, B2 8 mg/kg, niacin 45 mg/kg, pantothenic acid 15 mg/kg, B6 4 mg/kg, folic acid 1 mg/kg, B12 1 mg/kg, magnesium sulfate 53 mg/kg, choline chloride (60%) 500 mg/kg, zinc sulfate 2 mg/kg, manganese sulfate 18.5 mg/kg, copper sulfate 45 mg/kg, and ferrous sulfate 35 mg/kg.

**Table 3:** Ingredient composition and nutrient profile of finisher feeds (22–35 days).

Ingredients (%)	Treatments			
	Cont	LM-30	MM-60	HM-90
Corn	62.27	62.95	63.64	64.32
Canola meal	6.09	5.99	5.88	5.78
Soya bean meal	23.56	23.53	23.50	23.47
Soyabean oil	5.18	4.61	4.03	3.46
Monocalcium phosphate	0.51	0.51	0.51	0.51
Limestone	1.03	1.03	1.04	1.04
L-Lysine HCl	0.34	0.34	0.34	0.35
DL-Methionine	0.23	0.23	0.23	0.22
Threonine	0.06	0.06	0.06	0.06
β-mannanase	-	0.02	0.04	0.06
Sodium chloride	0.22	0.22	0.22	0.22
NaHCO_3_	0.20	0.20	0.20	0.20
VitaMin Premix *	0.30	0.30	0.30	0.30
Phytase	0.01	0.01	0.01	0.01
Total	100	100	100	100
ME (Kcal/kg)	3200	3170	3140	3110
Crude protein	19.50	19.50	19.50	19.50
Lysine	1.16	1.16	1.16	1.16
Methionine	0.47	0.47	0.47	0.47
Calcium	0.79	0.79	0.79	0.79
Available P	0.40	0.40	0.40	0.40

Cont (ME 3200 kcal/kg): without enzyme; LM-30 (ME −30 kcal/kg), MM-60 (ME −60 kcal/kg) and HM-90 (ME −90 kcal/kg) were supplemented with 200, 400, and 600 mg/kg β-mannanase, respectively. * Vitamin A 15,000 IU/kg, D3 3000 IU/kg, E 60 IU/kg, K3 3 mg/kg, B1 2 mg/kg, B2 8 mg/kg, niacin 45 mg/kg, B6 4 mg/kg, folic acid 1 mg/kg, pantothenic acid 15 mg/kg, B12 1 mg/kg, magnesium sulfate 53 mg/kg, choline chloride (60%) 500 mg /kg, zinc sulfate 2 mg/kg, manganese sulfate 18.5 mg/kg, copper sulfate 45 mg/kg, and ferrous sulfate 35 mg/kg.

**Table 4:** Effect of β-mannanase supplementation on growth performance in broilers fed low energy diets.

Items	Treatments				SEM
	Cont	LM-30	MM-60	HM-90	
Grower (2–3 weeks)					
Feed intake (g)	986.99	976.71	1012.42	984.95	7.69
Weight gain (g)	717.75	729.10	740.00	726.52	4.58
FCR	1.38	1.34	1.37	1.36	0.01
Finisher (4–5 weeks)					
Feed intake (g)	2179.01	2024.68	2074.38	2025.93	36.22
Weight gain (g)	1272.41	1142.64	1247.75	1192.17	29.04
FCR	1.72	1.78	1.67	1.70	0.02
Overall (2–5 weeks)					
Feed intake (g)	3167.02	3001.39	3086.80	3010.88	38.50
Weight gain (g)	1990.16	1871.73	1987.74	1918.68	28.71
FCR	1.59	1.60	1.55	1.57	0.01

Cont (ME 3100 kcal/kg): without β-mannanase; LM-30 (ME −30 kcal/kg), MM-60 (ME −60 kcal/kg) and HM-90 (ME −90 kcal/kg) were supplemented with 200, 400, and 600 mg/kg β-mannanase, respectively.

**Table 5:** Effect of β-mannanase supplementation on intestinal segments in broilers fed low energy diets.

Parameters	Treatments				SEM
	Cont	LM-30	MM-60	HM-90	
Weight (g)					
Duodenum	22.58	19.5 **	22.75	21.26	0.75
Jejunum	32.12	32.00	32.63	34.50	0.58
Ileum	24.42	19.20 ***	21.25 **	22.62	1.10
Ceca	11.81	11.62	11.50	10.88	0.20
Length (cm)					
Duodenum	14.67	14.32	15.02	14.60	0.14
Jejunum	63.10	75.46 ***	77.84 ***	77.00 ***	3.46
Ileum	64.24	61.68	58.8 **	58.32 ***	1.38
Ceca	17.86	18.18	16.60	16.80	0.39

Cont (ME 3100 kcal/kg): without β-mannanase; LM-30 (ME −30 kcal/kg), MM-60 (ME −60 kcal/kg) and HM-90 (ME −90 kcal/kg) were supplemented with 200, 400, and 600 mg/kg β-mannanase, respectively. The statistical difference from corresponding control value for a given parameter is indicated by: ** *p* < 0.01, *** *p* < 0.001.

**Table 6:** Effect of β-mannanase supplementation on meat quality traits in broilers fed low energy diets.

Parameters	Treatments				SEM
	Cont	LM-30	MM-60	HM-90	
Breast meat					
Shear force (g)	1026.01	973.05	874.88	1012.13	34.08
Cook loss (%)	26.40	27.17	25.56	26.86	0.35
WHC (%)	44.80	46.27	45.96	44.81	0.39
pH at 24 h	5.89 *	6.16	6.15	6.17	0.07
L	51.60	50.36	49.43	45.89 *	1.22
a	10.10	7.95	7.77	7.88	0.54
b	13.44	14.00	11.48	11.36	0.69
Thigh meat					
Cook loss (%)	32.02	33.28	33.51	32.26	0.39
WHC (%)	42.01	44.08	44.15	41.06	0.79
pH at 24 h	6.05	6.56 ***	6.42 **	6.48 ***	0.11
L	53.43	52.71	56.87	52.78	0.99
a	6.69	7.86	7.95	9.44	0.55
b	7.70	12.25	7.72	8.01	1.12

Cont (ME 3100 kcal/kg): without β-mannanase; LM-30 (ME −30 kcal/kg), MM-60 (ME −60 kcal/kg) and HM-90 (ME −90 kcal/kg) were supplemented with 200, 400, and 600 mg/kg β-mannanase, respectively. The statistical difference from corresponding control value for a given parameter is annotated by: * *p* < 0.05, ** *p* < 0.01, *** *p* < 0.001.

## Data Availability

Data can be obtained from the corresponding author upon reasonable request.

## References

[1] Dhawan S., Kaur J. (2007). Microbial mannanases: An overview of production and applications. Crit. Rev. Biotechnol..

[2] Chauhan P.S., Puri N., Sharma P., Gupta N. (2012). Mannanases: Microbial sources, production, properties and potential biotechnological applications. Appl. Microbiol. Biotechnol..

[3] Odetallah N.H., Ferket P.R., Grimes J.L., McNaughton J.L. (2002). Effect of mannanendo- 1,4-beta-mannosidase on the growth performance of turkeys fed diets containing 44 and 48% crude protein soybean meal. Poult. Sci..

[4] Anderson J.O., Warnick R.E. (1964). Value of enzyme supplements in rations containing certain legume seed meals or gums. Poult. Sci..

[5] Lee J.T., Bailey C.A., Cartwright A.L. (2003). β-Mannanase ameliorates viscosity associated depression of growth in broiler chickens fed guar germ and hull fractions. Poult. Sci..

[6] Duncan C.J., Pugh N., Pasco D.S., Ross S.A. (2002). Isolation of a galactomannan that enhances macrophage activation from the edible fungus Morchella esculenta. J. Agric. Food Chem..

[7] Jackson M.E., Geronian K., Knox A., McNab J., McCartney E. (2004). A dose-response study with the feed enzyme β-mannanase in broilers provided with corn-soybean meal based diets in the absence of antibiotic. Poult. Sci..

[8] Wu G., Bryant M.M., Voitle R.A., Roland D.A. (2005). Effet of beta-mannanase in corn-soy diets on commercial leghorns in second-cycle hens. Poult. Sci..

[9] Li Y., Zhang H., Chen Y.P., Yang M.X., Zhang L.L., Lu Z.X., Zhou Y.M., Wang T. (2015). Bacillus amyloliquefaciens supplementation alleviates immunological stress and intestinal damage in lipopolysaccharide-challenged broilers. Anim. Feed Sci. Technol..

[10] Jang A., Liu X.D., Shin M.H., Lee B.D., Lee S.K., Lee J.H., Jo C. (2008). Antioxidative potential of raw breast meat from broiler chicks fed a dietary medicinal herb extract mix. Poult. Sci..

[11] Hussein E.O.S., Suliman G.M., Alowaimer A.N., Ahmed S.H., Abd El-Hack M.E., Taha A.E., Swelum A.A. (2020). Growth, carcass characteristics, and meat quality of broilers fed a low-energy diet supplemented with a multienzyme preparation. Poult. Sci..

[12] Naqvi L.U., Nadeem A. (2004). Bioavailability of metabolizable energy through Kemzyme supplementation in broiler rations. Pak. Vet. J..

[13] Gunal M., Yasar S., Forbes J.M. (2004). Performance and some digesta parameters of broiler chickens given low or high viscosity wheat-based diets with or without enzyme supplementation. Turk. J. Vet. Anim. Sci..

[14] Mehri M., Adibmoradi M., Samie A., Shivazad M. (2010). Effects of β-Mannanase on broiler performance, gut morphology and immune system. Afr. J. Biotechnol..

[15] Chegeni A., Torki M., Kamyab A. (2011). Effects of β-mannanase-based enzyme in corn-soy and corn-soy-canola diets on broiler performance. J. Appl. Anim. Res..

[16] Sundu B., Kumar A., Dingle J. (2006). Response of broiler chicks fed increasing levels of copra meal and enzymes. Int. J. Poult. Sci..

[17] Saleh F., Tahir M., Ohtsuka A., Hayashi K. (2005). A mixture of pure cellulase, hemicellulase and pectinase improves broiler performance. Brit. Poult. Sci..

[18] Yu B., Wu S., Liu C., Gauthier R., Chiou P.W. (2007). Effects of enzyme inclusion in a maize-soybean diet on broiler performance. Anim. Feed Sci. Technol..

[19] Azarfar A. (2013). Effect of hemicell enzyme on the performance, growth parameter, some blood factors and ileal digestibility of broiler chickens fed corn/soybean-based diets. J. Cell Anim. Biol..

[20] Bedford M.R. (2000). Exogenous enzymes in monogastric nutrition-their current value and future benefits. Anim. Feed Sci. Technol..

[21] Wang Z.R., Qiao S.Y., Lu W.Q., Li D.F. (2005). Effects of enzyme supplementation on performance, nutrient digestibility, gastrointestinal morphology, and volatile fatty acid profiles in the hindgut of broilers fed wheat-based diets. Poult. Sci..

[22] Brenes A., Smith M., Guenter W., Marquardt R.R. (1993). Effect of enzyme supplementation on the performance and digestive tract size of broiler chickens fed wheat- and barley-based diets. Poult. Sci..

[23] Salim H., Kang H., Akter N., Kim D., Kim J., Kim M. (2013). Supplementation of direct-fed microbials as an alternative to antibiotic on growth performance, immune response, cecal microbial population, and ileal morphology of broiler chickens. Poult. Sci..

[24] Mohammadigheisar M., Kim H.S., Kim I.H. (2018). Effect of inclusion of lysolecithin or multi-enzyme in low energy diet of broiler chickens. J. Appl. Anim. Res..

[25] Bin Baraik B.S.S. (2010). Effect of Adding Xylanase and Phytase Enzymes to Broiler Diets on Performance and Carcass Yield and Quality. Ph.D. Thesis.

[26] Smith D., Lyon C., Lyon B. (2002). The effect of age, dietary carbohydrate source, and feed withdrawal on broiler breast fillet color. Poult. Sci..

